# Aspirin in the Food and Drug Administration Adverse Event Reporting System: Missing Demographics and Underreporting

**DOI:** 10.1055/s-0037-1606301

**Published:** 2017-08-30

**Authors:** Victor L. Serebruany, Ales Tomek, Moo Hyun Kim, Oleg Litvinov, Thomas A. Marciniak

**Affiliations:** 1 Division of Neurology, Johns Hopkins University, Baltimore, Maryland, United States; 2 Division of Neurology, Charles University and Motol Hospital, Prague, Czech Republic; 3 Division of Cardiology, Dong-A University, Busan, South Korea; 4 Bethany Beach, Delaware, United States

**Keywords:** aspirin, antiplatelet agents, adverse events, safety, registry

## Abstract

**Background** The U.S. Food and Drug Administration (FDA) Adverse Event (AE) Reporting System (FAERS) is a global passive surveillance repository requiring mandatory updates by pharmaceutical manufacturers. Oral antiplatelet agents (OAAs) including aspirin (acetylsalicylic acid [ASA]) are broadly used to prevent thrombosis, at the expense of extra bleeding risks. However, the OAA filing quality and their comparative patterns in FAERS are unknown. We assessed completeness of original annual FAERS reports for OAA with special attention on ASA.

**Methods** We extracted AE cases co-reported with OAA including ASA, clopidogrel, prasugrel, ticagrelor, vorapaxar, or their combination. The 2015 FAERS cases were examined based on OAA distribution, suspected causative role, missing gender or age, and most common AEs after ASA.

**Results** A total of 1,187,729 reports qualified the inclusion criteria. The majority ( *n* = 1,121,989) of the reports contain no reference of OAA, while 65,730 reports contain reference of at least one OAA, including 47,900 ASA cases. Therapy with ASA was heavily (>50%) underreported when used with prasugrel or ticagrelor, but still dominant (72.8%) among OAAs, followed by clopidogrel (18.7%), prasugrel (4.1%), ticagrelor (3.6%), and anecdotal vorapaxar (0.05%). Despite current recommendations, some (0.73%) reports contain multi-OAAs. The primary role of ASA in AE reporting was seldom (<1%), followed by clopidogrel (2.9%), prasugrel (3.7%), and highest for ticagrelor (9.3%). Missing gender after OAA was not common (<10%), but age was missing in approximately 25% of reports. Bleeding was the most frequent AE associated with ASA.

**Conclusion** The quality of reporting for OAA in general and ASA in particular can be improved by stricter FDA rules, better surveillance, and enforcements. Heavy ASA underreporting during dual antiplatelet therapy and missed demographic variables challenge outcome research capacities for establishing drug interactions in FAERS.

## Introduction

Being the most commonly used medicine in the world, aspirin shows benefits on myocardial infarction, stroke, and vascular death in numerous secondary prevention trials, and their meta-analyses. 1 2 With regard to the primary prevention, the totality of the evidence is less clear, as five published randomized trials with 55,000 apparently healthy individuals have far fewer endpoints than in the 194 studies of secondary prevention with 212,000 patients. 3 4 Overall, it seems that definite aspirin benefit for primary prevention is due solely to a reduction in first myocardial infarction of about one-third. 3 4 5 The data on stroke, vascular deaths, and affiliated bleeding remain inconclusive; so, further research particularly in the elderly population and diabetics will make important contributions to the existing totality of evidence. 1 2 3 4 5 6 Therefore, any further data yielded from the “real-life” large repositories or/and uniformed national registries are particularly useful.

The U.S. Food and Drug Administration (FDA) Adverse Event Reporting System (FAERS) is a database that contains information on adverse events and medication error reports submitted to the FDA. FAERS is a passive surveillance system that relies on voluntary reporting by health care professionals and consumers as well as mandatory reporting by pharmaceutical manufacturers. FAERS includes spontaneous reports from U.S. sources; serious and unlabeled spontaneous reports from non-U.S. sources, and serious, unlabeled, and attributable postmarketing clinical trial reports from all sources. 7 Data mining algorithms have been developed for the quantitative detection of signals from this vast database, that is, a signal means a statistical association between a drug and an adverse event. 8 Importantly, FAERS data are publicly available. 9

The randomized trials with all oral antiplatelet agents (OAAs) are commonly scarce on adverse events reporting, which is usually limited to the bleeding risks estimates. 1 2 3 4 Since posttrial safety data are not systematic, sources of comorbidities are entirely unknown, especially in the “real-life” clinical scenarios. Thus, evidence from large, uniform, government-mandated datasets is helpful to identify realistic patterns of OAA utilization and/or explore drug interactions with clinical outcomes. We here focus on annual (2015) aspirin cases reported to FAERS assessing quality and completeness of the submitted data.

## Methods

### Data Source

The FAERS reports originating in 2015 were qualified (which means that the initial report for the adverse event was dated in 2015), although the FDA received the report in the second quarter of 2016. There may have been follow-up reports associated with the same case after 2015, but the initial report was generated in 2015, and all repeated entries were disregarded. The pooled FAERS database was searched using the terms “aspirin,” “acetylsalicylic acid,” “clopidogrel,” “prasugrel,” “ticagrelor,” “vorapaxar,” “Plavix,” “Iscover,” “Zyllt,” “Effient,” “Efient,” “Brilinta,” “Brilique,” and “Zontibity” which were reported with an adverse event. Should the adverse event information was missing it was positioned into “Unknown” category.

### Outcomes

The primary endpoints of this study were distribution of major demographics (age and gender) and quality of reporting patterns for cases originating in 2015, the latest year for which all the records for aspirin, clopidogrel, prasugrel, ticagrelor, and vorapaxar have been updated in FAERS. To mitigate the issue of multiple reporting of a single event, originators were counted by unique case numbers rather than by report numbers. In lay terms, if a single case has four separate reports and each repeated report indicates different sources, the mandatory counting was a single first source, and three other upgrades were disregarded unless they add any previously missing information.

## Statistics

Analyses were done using Open VigilFDA v1.0.2, a web-based user interface for the FAERS database. This software allows for analysis of adverse drug events reported to the FDA. The reported adverse events can then be analyzed for “disproportionality” and scored using various measures of statistical significance. FAERS filing characteristics and other variables were compared among antiplatelet strategies. Categorical variables were estimated among OAAs using a chi-squared test, and continuous variables were compared using two-sample *t* -tests and nonparametric tests. The variables included were missed gender and age reporting, suspected causative role, and most common adverse events after acetylsalicylic acid (ASA). Statistical analyses were performed using SPSS version 13 (Chicago, Illinois, United States).

## Results

A total of 1,187,729 reports qualified the inclusion criteria. The majority ( *n* = 1,121,989) of the reports contain no reference of OAA, while 65,730 reports contain reference of at least one OAA, including 47,900 ASA cases. The annual distribution of OAA is detailed in Table 1 . Among all OAAs, aspirin has been reported most frequently, followed by clopidogrel, then equally for two latest P _2_ Y12 inhibitors, and only few anecdotal reports were issued on vorapaxar. Alarmingly, some multi-OAA adverse events on top of aspirin made the list, despite lack of any recommendations advocating for triple OAA. Another important fact is heavy (>50%) underreporting of aspirin use which is mandated for dual antiplatelet regimens concomitant with prasugrel or ticagrelor. Indeed, severe underreporting of aspirin use may be suspected with clopidogrel also; however, there are certain indications for monotherapy such as stroke/transient ischemic attack and peripheral artery disease, which are lacking for newer OAA. In fact, vorapaxar can be used with either aspirin or clopidogrel, but its utilization is anecdotal to assess and comprehend. The annual FAERS cases when aspirin is reported as a prime “suspect” or secondary agent for causing adverse event among other OAA regiments are outlined in Table 2 . The data in Table 2 indicate that very few (<1%) reports were suggestive of primary role of aspirin in FAERS. Importantly, causative impact of clopidogrel was lower (2.9%) than for prasugrel (3.7%), but especially frequently attributed to ticagrelor (9.3%). Secondary causative effect of OAA in general and aspirin in particular was dominant in the FAERS reports. Also notable and consistent were common aspirin underreporting and frequently applying aggressive triple antiplatelet strategies. With regard to the missing gender affiliated with OAA, these data are exhibited in Table 3 . Patients treated with aspirin have significantly less missing gender data (4.8%) than other patients (rest of the database; 113,605/1,139,818 [10.0%]), and this difference was significant ( *p* < 0.001). Only prasugrel reporting trended better than ASA. Interestingly, much fewer (993 [3.1%]) patients originated from the United States, compared with 1,317 (8.5%) non-U.S. patients who had missing gender data ( *p* < 0.001) for ASA. Another important issue is whether the ASA missing cases were mostly primary (the ones causing adverse event) or secondary (on top of another drug). These data with regard to age and gender and known role of ASA are exhibited in Table 4 . The data in Table 4 clearly indicate that demographics from most cases with identified role of ASA were predominantly missing. Moreover, 9,532 (29.5%) patients from the United States, compared with 3,185 (20.4%) non-U.S. patients, had missing age data ( *p* < 0.001). The most frequent adverse events associated with ASA are presented in Table 5 . As anticipated, different types of bleedings were the most common primary events attributed to aspirin.

**Table 1:** Overall annual (2015) distribution of OAA regimens reported in FAERS

Antiplatelet agent(s)	*N*	Any aspirin	No aspirin	Aspirin (%)
Aspirin only	47,900	47,900	0	100
Clopidogrel	12,284	6,217	6,067	51
Prasugrel	2,672	1,078	1,594	40
Ticagrelor	2,365	1,061	1,304	45
Vorapaxar	31	4	27	13
Two or more	478	293	185	61
None	1,121,989	0	1,121,989	0

Abbreviations: FAERS, Food and Drug Administration Adverse Event Reporting System; OAA, oral antiplatelet agent.

**Table 2:** Suspected causative role of antiplatelet strategies in FAERS in 2015

Antiplatelet regimen	*N*	Any aspirin	No aspirin	Aspirin (%)
Primary OAA/aspirin				
Aspirin only	454	454	0	100
Clopidogrel	362	174	188	48
Prasugrel	98	41	57	42
Ticagrelor	220	58	162	26
Vorapaxar	3	1	2	33
Two or more	28	15	13	54
Secondary OAA/aspirin				
Aspirin only	47,446	47,446	0	100
Clopidogrel	11,922	6,043	5,879	51
Prasugrel	2,574	1,037	1,537	40
Ticagrelor	2,145	1,003	1,142	47
Vorapaxar	28	3	25	11
Two or more	450	278	172	62

Abbreviations: FAERS, Food and Drug Administration Adverse Event Reporting System; OAA, oral antiplatelet agent.

**Table 3:** Reporting gender with OAA in FAERS

Antiplatelet regimen	Known gender	Missing gender
Aspirin only	45,591 (95.2%)	2,310 (4.8%)
Clopidogrel	11,329 (92.3%)	949 (7.7%)
Prasugrel	2,580 (96.6%)	91 (3.4%)
Ticagrelor	2,205 (93.3%)	159 (6.7%)
Vorapaxar	22 (71.0%)	9 (29.0%)
Two or more	438 (92.4%)	36 (7.6%)
None	1,009,639 (90.0%)	112,361 (10.0%)

Abbreviations: FAERS, Food and Drug Administration Adverse Event Reporting System; OAA, oral antiplatelet agent.

**Table 4:** Age and gender associated with the role of ASA reporting in FAERS

ASA role	Age known	Age unknown	Gender known	Gender unknown
Primary suspect	325 (62.9%)	192 (37.1%)	445 (86.1%)	72 (13.9%)
Secondary suspect	417 (71.4%)	167 (28.6%)	494 (84.6%)	90 (15.4%)
Interacting	29 (82.9%)	6 (17.1%)	32 (91.4%)	3 (8.6%)
Concomitant	6,632 (71.0%)	2,707 (29.0%)	9,050 (96.9%)	289 (3.1%)
Unknown	27,781 (74.2%)	9,645 (25.8%)	35,570 (95.0%)	1,856 (5.0%)

Abbreviations: ASA, acetylsalicylic acid; FAERS, Food and Drug Administration Adverse Event Reporting System.

**Table 5:** Most common adverse events after aspirin in FAERS (2015)

Adverse event	Patients (%)
Bleeding	6,756 (14.1%)
GI bleeding	3,302 (6.9%)
Intracranial bleeding	717 (1.5%)
Anemia	2,314 (4.8%)
Dyspnea	2,286 (4.8%)
Myocardial infarction	798 (1.7%)
Stroke	1,422 (3.0%)
Acute coronary syndrome	860 (1.8%)
Thrombosis	1,015 (2.1%)
Thrombosis in device	48 (0.1%)
Arrhythmia	1,494 (3.1%)
Ventricular arrhythmia	209 (0.4%)
Torsade	36 (0.1%)
Angioedema	708 (1.5%)

Abbreviations: FAERS, Food and Drug Administration Adverse Event Reporting System; GI, gastrointestinal.

## Discussion

Data from this large, uniform, U.S. government–run international registry revealed poor reporting quality for OAA in general and aspirin in particular. It seems that better FAERS monitoring implying stricter rules and enforcements is warranted. Our data indicate heavy aspirin underreporting during dual antiplatelet therapy, and missed demographic variables. These shortcomings challenge quality of outcome research and establishing drug interactions with adverse events applying FAERS data.

Among all modern antiplatelet strategies, aspirin still remains the cornerstone with the broadest possible utilization. Aspirin irreversibly acetylates a serine residue at position 530 on the cyclooxygenase (COX) enzyme, thus inhibiting the first step in the transformation of arachidonic acid to the platelet agonist thromboxane A _2_ , a powerful promoter of aggregation. 10 The irreversible nature of COX inhibition underlies the ability of low doses of aspirin administered chronically, to inhibit platelet aggregation in vivo. 11 There is a nonlinear relationship of inhibition of platelet thromboxane A _2_ generation with inhibition of thromboxane-mediated platelet aggregation, requiring in excess of 95% inhibition to influence function. 12 Importantly, most antiplatelet strategies include aspirin as a back-up for newer agents; therefore, any analyses of clinical outcomes associated with patented OAA will not be possible, unless the precise role of aspirin is clearly identified. In fact, our previous experience with FAERS suggests poor quality of event reporting. 13 Clopidogrel monotherapy is common post-stroke, in contrast to prasugrel and ticagrelor which must be used with ASA. Therefore, it was a quality test for FAERS, as prasugrel and ticagrelor cases should mandatorily co-report ASA. However, these data were heavily missed from FAERS.

There are few important considerations which may be yielded from the index data. Indeed, the quality of FAERS reports was similarly average for all antiplatelet agents. There is nothing unique about aspirin reporting quality, and all OAA data are suffering from missing entries. More missing data after aspirin are probably attributed to much larger sample size, and domination among all antiplatelet-associated adverse events. Alarmingly high rate of nonreported gender in over a quarter of cases is unacceptable. Massive missed or/and unknown demographics preclude from better understanding of the drug safety profiles, somewhat challenging the entire idea behind FAERS. Especially concerning is a high incidence of adverse event reporting during triple antithrombotic strategies. This segment should be under close monitoring because novel oral anticoagulants will broadly supplement conventional dual antiplatelet strategies. Since the FDA mandates and oversees this valuable huge repository keeping it public, improving the quality of reports should be the upmost priority. In fairness, aspirin reporting may be tricky as there are numerous local manufacturers not complying with the FAERS reporting laws. Such an “orphan” status of aspirin may be partially responsible for the filing failures. Nevertheless, this study has important practical implications. First and upmost, the quality of the FAERS aspirin reporting is unacceptable, raising concerns about other drugs. Considering that U.S. filing is far better than reports around the globe, 13 it seems the FDA should consider better options to stimulate proper international reporting, potentially switching such responsibility to the consumers or health care professionals away from manufacturers. Acknowledging sharp decline in ongoing or planned clinical trials with antiplatelet agents, the “real-life” data from FAERS are definitely useful if properly managed. Since FAERS is public, any scientist may access the data and mine this huge repository. Moreover, FAERS maintenance is paid by U.S. tax dollars, requiring definite optimization and better surveillance.

There are obvious strengths in our approach with this study. This analysis was conducted within the frame of a government database, requiring mandatory serious event reporting. 14 The data retrieval and analyses were done by one of the authors (T.A.M.) with decades of experience working for the FDA. The sample size was sufficient to make reasonable conclusions on filing quality. Our study also has some limitations. The FAERS database analyses are always challenged by the often uneven mixture of patients and reports, since any single event can generate multiple records. Another shortcoming is that FAERS applies complicated accounting, making statistical claims for common adverse events challenging. There are also no mandatory deadlines for updates required by and strictly forced by the FDA; therefore, some data may still be missing or delayed. Another disadvantage is that we did not encompass the entire database, allowing the in-depth examination of the totality of the extracted evidence, but limited our work to the most recent full year (2015) for which the FAERS data are available. Further research should expand demographics beyond age and gender, concomitant use of proton pump inhibitors, and explore the entire FAERS data, not limited by the annual reports. The causative impact was greater for ticagrelor than for prasugrel or clopidogrel. This finding may reflect how long the drugs have been around, as enthusiasm for reporting AEs may fade over time. Regardless, the consistency and magnitude of the observed differences suggest that the index data are realistic, and should not be disregarded. The FDA should enforce quality and completeness of aspirin reports for better surveillance.
